# Target localization using scanner‐acquired SPECT data

**DOI:** 10.1120/jacmp.v13i3.3724

**Published:** 2012-05-10

**Authors:** Justin R. Roper, James E. Bowsher, Joshua M. Wilson, Timothy G. Turkington, Fang‐Fang Yin

**Affiliations:** ^1^ Department of Radiation Oncology Duke University Medical Center Durham NC 27710 USA; ^2^ Department of Radiology Duke University Medical Center Durham NC 27710 USA

**Keywords:** single photon emission computed tomography, target localization, radiation therapy delivery

## Abstract

Target localization using single photon emission computed tomography (SPECT) and planar imaging is being investigated for guiding radiation therapy delivery. Previous studies on SPECT‐based localization have used computer‐simulated or hybrid images with simulated tumors embedded in disease‐free patient images where the tumor position is known and localization can be calculated directly. In the current study, localization was studied using scanner‐acquired images. Five fillable spheres were placed in a whole body phantom. Sphere‐to‐background ${\;}^{99m}\text{Tc}$ radioactivity was 6:1. Ten independent SPECT scans were acquired with a Trionix Triad scanner using three detector trajectories: left lateral 180°, 360°, and right lateral 180°. Scan time was equivalent to 4.5 min. Images were reconstructed with and without attenuation correction. True target locations were estimated from 12 hr SPECT and CT images. From the 12 hr SPECT scan, 45 sets of orthogonal planar images were used to assess target localization; total acquisition time per set was equivalent to 4.5 min. A numerical observer localized the center of the targets in the 4.5 min SPECT and planar images. SPECT‐based localization errors were compared for the different detector trajectories. Across the four peripheral spheres, and using optimal iteration numbers and postreconstruction smoothing, means and standard deviations in localization errors were 0.90±0.25 mm for proximal 180° trajectories, 1.31±0.51 mm for 360° orbits, and 3.93±1.48 mm for distal 180° trajectories. This rank order in localization performance is predicted by target attenuation and distance from the target to the collimator. For the targets with mean localization errors < 2 mm, attenuation correction reduced localization errors by 0.15 mm on average. The improvement from attenuation correction was 1.0 mm on average for the more poorly localized targets. Attenuation correction typically reduced localization errors, but for well‐localized targets, the detector trajectory generally had a larger effect. Localization performance was found to be robust to iteration number and smoothing. Localization was generally worse using planar images as compared with proximal 180° and 360° SPECT scans. Using a proximal detector trajectory and attenuation correction, localization errors were within 2 mm for the three superficial targets, thus supporting the current role in biopsy and surgery, and demonstrating the potential for SPECT imaging inside radiation therapy treatment rooms.

PACS numbers: 87.55.Gh, 87.57.qp, 87.57.uh

## I. INTRODUCTION

Single photon emission computed tomography (SPECT) is a functional and molecular imaging modality, which is being used clinically to localize targets prior to biopsy and surgery.^(^ ^1^ ^–^ ^5^ ^)^ SPECT is also being investigated for imaging inside radiation therapy treatment rooms in an effort to realize the potential of biologically conformal radiation therapy.^(^ ^6^ ^,^ ^7^ ^)^ In these applications, the accuracy and precision of SPECT localization is key. Previous studies on SPECT localization have used computer‐simulated or hybrid images with simulated tumors embedded in disease‐free patient images. In such simulation studies, the tumor position is then known and localization can be calculated directly.^(^ ^6^ ^–^ ^10^ ^)^ Computer simulations, however, only approximate imaging with a real detector. For clinical SPECT systems, image quality can be degraded by factors such as mechanical misalignments and imprecise calibration, which are not typically included in simulations. As such, it is important to quantitatively evaluate localization in scanner‐acquired SPECT images to gauge the level of performance that could be achieved clinically.

Many factors can influence the detector trajectory. For example, the trajectory may be altered by immobilization devices and other equipment, or by patient positioning considerations. For on‐board imaging during radiation therapy, in which the target position is known approximately prior to imaging, the detector trajectory might be modified to improve sensitivity and spatial resolution in the target region. Because detector trajectories vary, it is important to understand how localization performance depends on detector trajectory.

Localization may be affected by other factors, as well. For example, the influence of scatter correction has been considered in the detection‐localization of hot spheres.^(^ ^8^ ^)^ Other aspects of SPECT image reconstruction may also influence localization. In the present study, localization was evaluated using scanner‐acquired planar and SPECT images of hot spheres that were positioned deep and superficial in a whole‐body phantom. Targets were localized using a numerical observer and compared to the true locations, which were measured with CT and a high‐count SPECT study. SPECT‐based localization performance was compared for proximal 180°, distal 180°, and 360° detector trajectories to determine the effect of the detector trajectory on localization errors. Localization accuracy was evaluated as a function of attenuation correction, image‐reconstruction iteration number, and postreconstruction smoothing.

## II. MATERIALS AND METHODS

### A. Phantom

**Figure 1:**
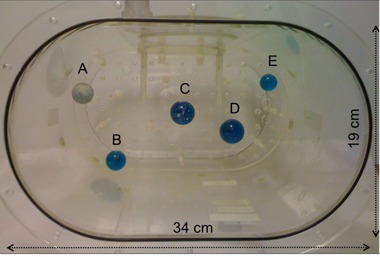
Oval phantom with 5 spheres of 6:1 ${\;}^{99m}\text{Tc}$ activity ratio relative to background.

Five fillable spheres — targets labeled A–E — were placed in a single 40 cm section of the Extended Oval PET Phantom (Data Spectrum, Hillsborough, NC) as shown in Fig. 1. The inner dimensions of the phantom are 34 cm laterally and 19 cm anterior–posteriorly, with a 1 cm thick Acrylic wall.^(^ ^11^ ^)^ The spheres were weighed before and after filling, and by assuming that the radioactive solution had a density of $1\;g/{\text{cm}}^{3}$, the inner diameters of targets A–E were calculated as 28, 28, 34, 34, and 22 mm. The target‐to‐background ${\;}^{99m}\text{Tc}$ radioactivity ratio was 6:1 and is based on clinically observed tumor‐to‐background activity uptake in breast cancer imaging.^(^ ^12^ ^)^ Background radioactivity was approximately 0.52 μCi/mL at the time of the first SPECT scan.

### B. Imaging

**Figure 2:**
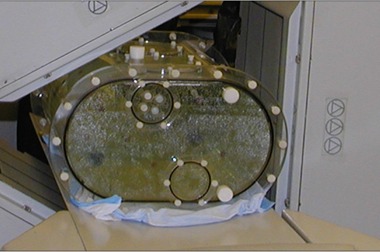
One detector of a Trionix Triad SPECT scanner was used to image the phantom.

As shown in Fig. 2, the phantom was imaged using one detector of a Trionix Triad SPECT scanner [Trionix Research Laboratory, Twinsburg, OH] that was equipped with low‐energy, ultrahigh resolution (LEUR) parallel hole collimation. The LEUR collimator holes were 1.4 mm in diameter by 34.9 mm in length. A photopeak window for ${\;}^{99m}\text{Tc}$ was centered on 140 keV ± 10.0%. Projection images were acquired every 2° in step‐and‐shoot mode and implemented on a 256 × 128 grid of $1.78\;\times \;1.78\;{\text{mm}}^{2}$ bins. In regions of the detector illuminated by background activity, approximately seven photons were detected in the photopeak window every second per ${\text{cm}}^{2}$. The scan time was adjusted as the radioactivity decayed to maintain the equivalent of a 4.5 min clinical scan for a background activity level of 0.25 μCi/mL. The modeled scan time does not include the time spent rotating the detector and, thus, models the number of counts expected from list‐mode acquisition.

**Figure 3:**
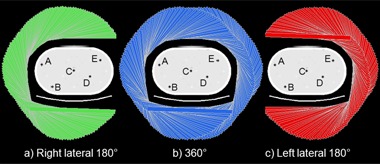
Three detector trajectories were each used to acquire 10 independent, 4.5 min SPECT scans.

Three noncircular detector trajectories were investigated — left lateral 180°, 360°, and right lateral 180° (as illustrated in Fig. 3) — that contour to the phantom and couch. There was no transaxial truncation of phantom activity at any detector view. Scan time was equivalent for each trajectory. The 360° trajectory had twice the number of views yet half the count time per view as compared with the 180° trajectories. For each trajectory, an ensemble of 10 independent scans was acquired. SPECT images were reconstructed on a grid of $1.78\;\times \;1.78\;\times 1.78\;{\text{mm}}^{3}$ voxels using MLEM^(^ ^13^ ^,^ ^14^ ^)^ both with and without corrections for attenuation. Images for 1–10 iterations were smoothed after reconstruction with a series of 3D Gaussian filters that ranged in FWHM from 0 to 22.5 mm in increments of 2.5 mm. The attenuation map was derived from a CT image acquired at 120 kVp on a GE Lightspeed scanner (General Electric, Waukesha, WI). CT voxels were $0.9766\;\times \;0.9766\;\times \;1.250\;{\text{mm}}^{3}$.

To obtain low‐noise images for estimating true target positions, the phantom was then scanned for 12 hrs using the 360° orbit described above. Scatter data were collected in a window set at 103 ±17.5% keV. The 12 hr SPECT was corrected for attenuation and spatially‐varying spatial resolution during reconstruction using 10 subset OSEM. The 10 subsets were used because of slower convergence due to spatial resolution modeling.^(^ ^15^ ^)^ Iterations 1–10 were recorded.

### C. True target positions

**Figure 4:**
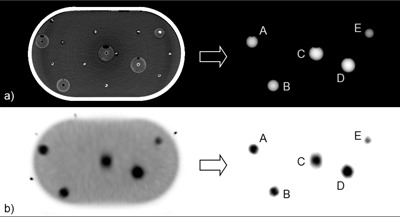
The positions of targets A–E were derived from (a) CT and (b) 12 hr SPECT images and were used subsequently to calculate localization error in the 4.5 min SPECT images. For display purposes, these images have been summed over axial slices containing a target.

To calculate localization error, the true position of a target must be known. In this study, the true positions of the targets were estimated from the 12 hr SPECT and CT images. In the 12 hr SPECT image, ROIs were placed on targets A–E. Background activity was subtracted, and voxels with intensities greater than a nominal fraction of the maximum ROI value were used in a center of mass calculation. These calculations of target centroids were performed for iterations 1–10. To evaluate these estimates, SPECT‐based target positions were registered with those from the CT using Horn's method,^(^ ^16^ ^)^ and residual errors were calculated. In the CT image, a target position was defined as the centroid of the water inside a sphere. Figure 4 shows the CT and 12 hr SPECT images, summed over axial slices containing a target, and the corresponding images of the targets.

### D. Attenuation and distance

**Figure 5:**
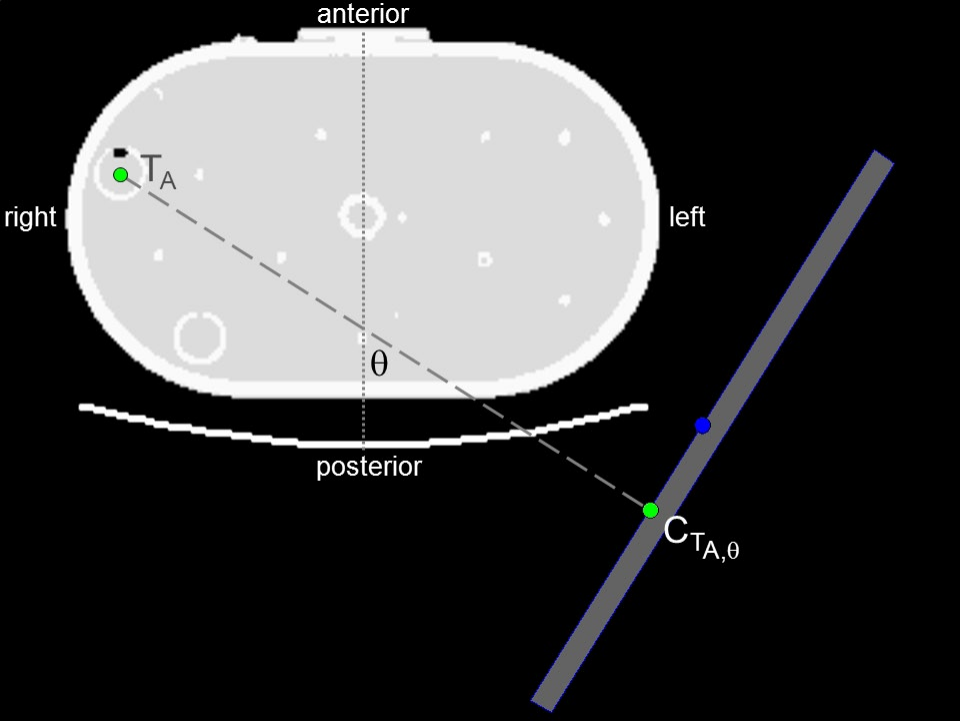
The distance between a target and the collimator at detector angle θ. Attenuation was also calculated along the line segment.

Target attenuation and distance to the collimator were calculated for all detector views in each detector trajectory. These metrics were used in subsequent comparisons of localization performance across the different detector trajectories. To estimate the attenuation of 140 keV photons, the CT image was segmented into air, water, and Acrylic with linear attenuation coefficients of 0.000, 0.155, and $0.176\;{\text{cm}}^{-1}$, respectively. Although not imaged by CT, the aluminum SPECT couch — approximately 0.3 cm thick, 35 cm wide, and 3 cm deep — was visible in the 12 hr scatter‐window SPECT image. This image was used to estimate the couch position. The SPECT couch was assigned a linear attenuation coefficient of $0.3895\;{\text{cm}}^{-1}$. At each detector view θ, attenuation survival probability $P_{\text{Ti},\theta }$ was calculated along a central ray between the center of a target $T_{i}$ and the collimator location $C_{\text{Ti},\theta }$. $P_{\text{Ti},\theta }$ is the fraction of photons that are not attenuated by the phantom or couch and is described mathematically as follows:

(1) $$P_{T_{i},\theta }=e^{-\int_{T_{i}}^{C_{T_{i,\theta }}}\mu \;dr}$$

where *i* is an index of targets A–E, and μ is the linear attenuation coefficient over a line segment *dr*. Figure 5 shows the central ray between target A and the collimator at detector angle θ. Collimator spatial resolution is degraded in proportion to the distance between a target and collimated detector. Collimator‐target distance $R_{\text{Ti},\theta }$ was calculated at every detector angle θ for targets A–E:

(2) $$R_{T_{i,\theta }}=\sqrt{{\left(x_{T_{i}}-x_{C_{T_{i,\theta }}}\right)}^{2}+{\left(y_{T_{i}}-y_{C_{T_{i,\theta }}}\right)}^{2}}$$

### E. SPECT target localization

Target localization was assessed in the 4.5 min equivalent SPECT scans using numerical observers. The observers were forced to select the most suspicious target location. Observer templates were binary spheres of the same diameters as the targets. As such, the templates were not matched exactly to the targets in the SPECT images because the targets appeared blurred in the reconstructed images, with apparent diameters greater than true diameters. An observer scanned each search volume (Ω), a 36 mm diameter sphere that was centered on a target. The size of Ω was designed to include positioning uncertainties common to radiation therapy delivery. Each Ω was sampled at every voxel, and the voxel with the highest normalized cross‐correlation value and its 26 neighbors were then sampled at every 1/3 voxel width. The measured target position *M* was the subvoxel with the highest normalized cross‐correlation value λ between the observer template *w* and the image *g*, which is defined mathematically as:

(3) $$\lambda_{i,k}=\frac{w_{i}\cdot g_{i,k}}{\sqrt{\left(w_{i}\cdot w_{i})(g_{i,k}\cdot g_{i,k}\right)}}$$

(4) $$M_{i}(x,y,z)=\text{arg}\;\underset{k\in \Omega_{i}}{\text{max}}(\lambda_{i,k})$$

where *i* indexes targets A–E, *k* indexes the centers of subvoxels within $\Omega_{i}$, and $\Omega_{i}$ is the search volume about target *i*. Localization error was calculated as the Euclidean distance *dr* between the measured *M* and true *T* target positions:

(5) $$dr_{i}=\sqrt{{\left(x_{M_{i}}-x_{T_{i}}\right)}^{2}+{\left(y_{M_{i}}-y_{T_{i}}\right)}^{2}+{\left(z_{M_{i}}-z_{T_{i}}\right)}^{2}}$$

Localization errors were recorded for every combination of smoothing and iteration number. For each of these combinations, mean localization error was calculated across the 10 independent SPECT images. The worst‐case, maximum localization error was also noted. The distributions of mean and maximum localization errors were displayed using box plots to show the variation in localization across different iteration numbers and degrees of smoothing.

The combination of iteration number and smoothing was recorded that yielded the lowest mean localization error. Optimal combinations of smoothing and iteration number were used in subsequent assessments of localization performance between detector trajectories and reconstructions with and without attenuation correction. Box plots were used to show the distribution of localization errors attributable to quantum noise.

### F. Planar target localization

**Figure 6:**
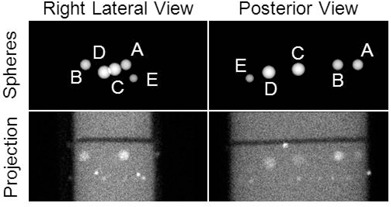
Target positions are shown above respective planar images from orthogonal detector views. In planar images, targets are more clearly visible at a view of closer approach and minimal attenuation. The small regions of intense activity are fiducial markers.

Target localization was investigated using orthogonal pairs of planar images. From the 12 hr SPECT scan, planar images with sufficiently high numbers of counts were thinned by a binomial function to simulate 4.5 min in total acquisition time, which was divided equally between the two detector views. In total there were 45 sets of orthogonal planar images spanning 180° posteriorly.^(^ ^17^ ^)^ Similar to the localization methods used in the SPECT study, localization was assessed using a numerical observer. However, localization was performed using 2D images and observer templates. At each detector view, a 2D template was derived by forward projecting a 3D template onto the image plane; background activity and attenuation were not modeled. Templates were then cross‐correlated across 36 mm diameter circular search regions surrounding the respective spheres. Locations with the highest values were used as estimates of target positions. From the pair of orthogonal images, the detector view of closer approach was used exclusively for longitudinal localization. Localization errors were calculated using Eq. (5). Figure 6 shows superimposed 2D templates above a set of planar images.

## III. RESULTS

### A. Reconstructed images

**Figure 7:**
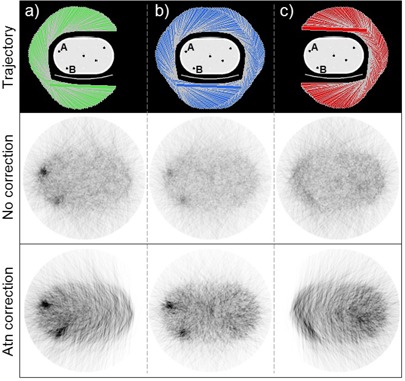
Images from the 4.5 min SPECT scans are displayed beneath their respective detector trajectories. Rows separate images reconstructed with and without attenuation correction. This particular axial slice includes targets A and B.

Figure 7 shows SPECT images reconstructed using data from the three detector trajectories: right lateral 180°, 360°, and left lateral 180°; the images are from the second iteration. This axial slice includes targets A and B. Because of limited spatial resolution, the apparent target‐to‐background activity ratio in these images is less than the 6:1 activity ratio of the phantom. The target signal is strongest in images from the right lateral 180° detector trajectory and weakest in the left lateral 180° images, thus showing the effects of the detector trajectory on reconstructed images.

### B. Registration

**Figure 8:**
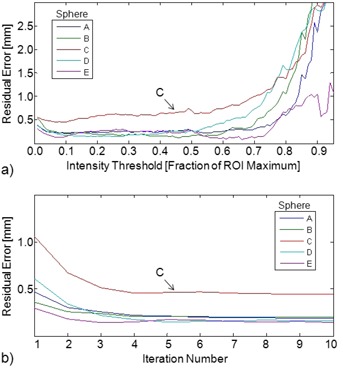
The residual errors in the registration of target positions derived from CT and 12 hr SPECT images: (a) at SPECT iteration number 4, residual errors are plotted as a function of the intensity threshold used to differentiate the target from background; (b) Using a fixed intensity threshold of 0.1, residuals errors are plotted for iterations 1–10.

The true positions of targets A–E were estimated from 12 hr SPECT and CT images. The sum of residual errors was minimized using SPECT‐based position estimates from iteration number 4 with an intensity threshold of 10% of each ROI maximum value. The residual errors were 0.22, 0.21, 0.45, 0.18, and 0.15 mm for targets A–E. Plots in Fig. 8 show that residual errors were similar over a broad range of iteration numbers and intensity thresholds.

### C. Attenuation and distance

**Figure 9:**
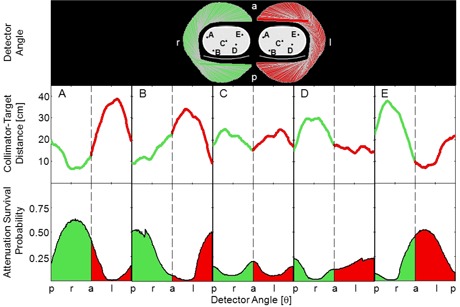
Collimator‐target distance and photon attenuation survival probability are plotted as a function of detector angle. These metrics are related to spatial resolution and noise in the target signal. Results are color‐coded according to the detector trajectory. Abrupt changes in attenuation survival probability, most noticeable in B and D, correspond to edges of the couch.

For targets A–E, the attenuation survival probability and distance to the collimator were calculated at each detector angle using Eqs.(1) and (2). Results are summarized in Fig. 9 by two sets of curves. The top row shows the distance between the collimator and a target. Smaller values indicate better spatial resolution. A series of filled curves in the bottom row show the fraction of photons originating from the center of a target that are not attenuated by the phantom or SPECT couch. Larger area under the filled curve indicates that a greater fraction of target photons are detected. Right and left lateral 180° trajectories were divided at anterior and posterior angles. Distance and attenuation results displayed in Fig. 9 were averaged over all angles for each detector trajectory and are reported in Table 1. Values reported for the 360° trajectory are the average of the left and right lateral 180° trajectories. Target C was centered in the phantom such that the mean attenuation survival probability and distance to the collimator were equivalent for each trajectory. For targets A, B, D, and E, proximal 180° trajectories yielded the lowest collimatortarget distances and the highest photon survival probabilities, followed by 360° and then distal 180° trajectories. Distance and attenuation were best for proximal 180° trajectories.

**Table 1:** Attenuation survival probability and the distance between a target and the collimator are reported for targets A–E, as averaged over all views in a detector trajectory.

		*Mean Target‐Collimator Distance (cm)*			*Mean Attenuation Survival Probability*	
*Target*	*R 180°*	*360°*	*L 180°*	*R 180°*	*360°*	*L 180°*
A	11	21	30	0.51	0.31	0.11
B	14	21	27	0.34	0.25	0.17
C	21	21	21	0.10	0.10	0.10
D	26	21	16	0.09	0.13	0.18
E	29	21	13	0.14	0.26	0.38

### D. SPECT target localization

**Figure 10:**
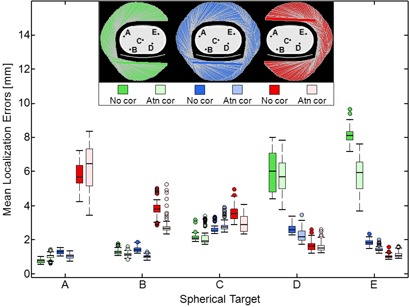
Box plots show the distribution of mean localization errors using the various smoothing filters at iteration numbers (2–10). The first iteration was excluded because of numerous outlying data points. Tightly clustered data indicates that iteration number and smoothing do not strongly affect target localization.

Target localization was assessed in 4.5 min SPECT images from three detector trajectories, which were reconstructed with and without attenuation correction. Each box plot in Fig. 10 represents the spread in the a) mean and b) maximum localization errors across the 90 combinations of 2–10 iterations and the 10 degrees of smoothing. The boxes are color‐coded according to detector trajectory and grouped by target site A–E. The median is marked by a horizontal line within the interquartile range (IQR). Whiskers extend from the IQR to the last data point within 1.5 times the IQR. Outliers from iterations 2–10 are marked by filled circles. Because of numerous outliers, the first iteration was not considered in this analysis. These results are useful for gauging the robustness of target localization to postreconstruction smoothing and iteration number. The effects of smoothing and iteration number on mean localization error, shown in Fig. 9, are summarized by means and standard deviations in Table 2.

**Table 2:** Mean localization error was calculated at iterations 2–10 using different degrees of smoothing and are summarized here by a mean and standard deviation. A small standard deviation indicates that target localization was not strongly affected by smoothing or iteration number.

			*Mean (mm)*		*Standard Deviation (mm)*		
*Target*	*Recon*	*R 180°*	*360 °*	*L 180°*	*R 180°*	*360°*	*L 180°*
A	No Cor	0.75	1.28	5.76	0.12	0.13	0.80
	AtnCor	1.02	1.03	6.18	0.17	0.14	1.36
B	No Cor	1.28	1.42	3.77	0.13	0.15	0.48
	AtnCor	1.14	1.02	2.82	0.14	0.10	0.54
C	No Cor	2.15	2.66	3.61	0.21	0.29	0.49
	AtnCor	2.12	2.84	2.98	0.44	0.33	0.52
D	No Cor	5.97	2.65	1.63	1.15	0.30	0.28
	AtnCor	5.68	2.26	1.56	0.99	0.36	0.27
E	No Cor	8.17	1.83	1.02	0.41	0.17	0.11
	AtnCor	5.81	1.45	1.13	1.00	0.15	0.18

Listed in Table 3 are the iteration numbers and smoothing values that yielded the lowest mean localization errors for each target, reconstruction method, and detector trajectory. Subsequent comparisons of localization errors are made using these optimal parameters.

**Table 3:** The iteration number and degree of postreconstruction smoothing were recorded that yielded the lowest mean localization error for each detector trajectory and reconstruction method.

			*Iteration Number*		*Gaussian Smoothing FWHM (mm)*			*Mean Localization Error (mm)*		
*Target*	*Recon*	*R 180°*	*360°*	*L 180°*	*R 180°*	*360°*	*L 180°*	*R 180°*	*360°*	*L 180°*
A	No Cor	4	2	4	5.0	22.5	17.5	0.5	1.0	4.2
	AtnCor	2	3	1	2.5	5.0	22.5	0.6	0.8	3.0
B	No Cor	4	4	5	7.5	7.5	5.0	1.1	1.2	2.8
	AtnCor	4	3	4	5.0	7.5	0.0	0.8	0.8	2.3
C	No Cor	8	1	10	7.5	20.0	20.0	1.8	2.1	2.8
	AtnCor	3	1	8	12.5	22.5	22.5	1.7	2.1	2.3
D	No Cor	9	6	9	22.5	10.0	10.0	4.4	2.3	1.2
	AtnCor	8	9	7	20.0	10.0	15.0	3.8	1.7	1.2
E	No Cor	1	3	1	0.0	10.0	5.0	7.2	1.5	0.8
	AtnCor	3	3	3	22.5	7.5	7.5	3.7	1.2	0.9

**Figure 11:**
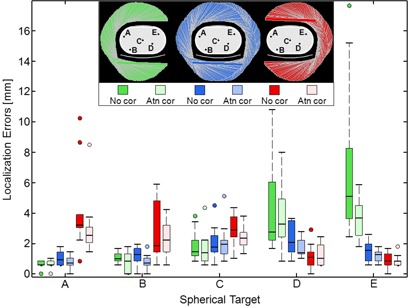
Localization errors from the 4.5 min SPECT scans are summarized by box plots, which are grouped by target site A–E and color‐coded by detector trajectory. Horizontal lines mark medians. Outliers are shown with circles.

Localization was evaluated for the different detector trajectories. Localization errors were calculated across the 10‐image ensemble using Eqs. (3) to (5). The distribution of localization errors are displayed in Fig. 11 using box plots. These plots show the spread in localization errors due to quantum noise. For right‐sided target A, localization errors were the lowest using a right lateral 180° trajectory, were slightly higher with the 360° trajectory, and were the highest and substantially so with the left lateral 180° trajectory. Similarly, for left‐sided targets D and E, localization errors were lowest using the left lateral 180° trajectory and were highest for the right lateral 180° trajectory. These results show the benefit of using a proximal 180° trajectory.

Localization was compared for images reconstructed with and without attenuation correction. For the targets with mean localization errors < 2 mm, attenuation correction reduced localization errors by 0.15 mm on average. The improvement was 1.0 mm on average for the more poorly localized targets.

Localization was analyzed across different iteration numbers and degrees of postreconstruction smoothing. With a proximal 180° trajectory and attenuation correction, the mean localization errors averaged across 10 SPECT images for four peripheral spheres were all within 1.2, 1.6, and 2.6 mm, respectively, using the best, average, and worst combinations of smoothing and iteration number. The effects of smoothing and iteration number were also analyzed for the worst‐case localization errors — the maximum across the 10 SPECT images. Using the best, average, and worst combinations of smoothing and iteration number, the worst‐case localization errors for the superficial spheres were within 1.7, 2.2, and 2.9 mm, respectively. Many of the largest localization errors were from the first iteration.

### E. Planar target localization

**Figure 12:**
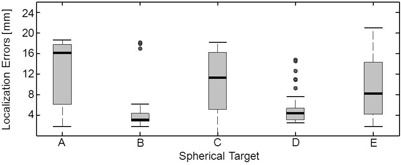
Planar‐based localization errors summarized using box plots from 45 sets of orthogonal detector views.

Planar‐based localization estimates from 45 sets of orthogonal detector views are summarized in Fig. 12. Target localization was typically better for the more posteriorly located spheres B and D as compared with the other spheres because, on average, the detector was closer and attenuation less severe. Localization performance for a particular sphere varied widely across detector views. In the best‐case scenario, localization error was within 2.5 mm for all spheres. However, mean localization errors ranged from 4.7 to 12 mm and in the worst cases, these errors were approximately 20 mm.

## IV. DISCUSSION

For nuclear medicine imaging tasks in which localization is important, it is necessary to understand the associated error in the localization. One such task is biopsy where localization uncertainties might be used in part to develop sampling strategies. Another use — and the broader goal that this study is a component of — is using imaging in radiation therapy delivery for more accurate patient positioning to reduce margins, which may be ≤ 3 mm for stereotactic procedures or ≥ 10 mm in treatments where uncertainties are greater.^(^ ^18^ ^–^ ^20^ ^)^ Toward this application, all SPECT‐based localization errors in this study were ≤ 2 mm for the superficial targets when using optimal reconstruction parameters and proximal trajectories. For targets such as these, it may be that SPECT, in combination with already available X‐ray imaging, could be used to improve localization. Just as CT complements functional information from SPECT in the diagnostic setting, high resolution cone‐beam CT imaging in the treatment room would provide much of the information on normal structures, external boundaries, and markers.

Targets for biopsy, surgery, or radiation therapy may be smaller than 1 cm in width or much larger than those evaluated in this study. In a previous study, we investigated localization performance for hot spheres ranging in diameter from 11 to 22 mm.^(^ ^6^ ^)^ In light of challenges observed in localizing the 11 mm targets, we are investigating the use of multipinhole SPECT to better image a relatively small volume surrounding a target. Though localization was not assessed, targets of 7 to 10 mm in diameter were often markedly better visualized in multipinhole than in parallel‐hole–collimated SPECT images.^(^ ^21^ ^)^ The present study expands on our earlier work by considering spherical targets of 22 to 34 mm in diameter and the use of a real parallel‐hole SPECT imaging system. Similarly there are wide variations in tumor‐to‐background uptake ratios that depend on the radiotracer and patient. That noted, the 6:1 uptake ratio used in this study is in the range of those observed in patient studies with SPECT imaging for a variety of malignancies.^(^ ^12^ ^,^ ^22^ ^–^ ^24^ ^)^ The evaluation of localization performance over a range of sizes and uptake ratios is important for determining clinical scenarios in which SPECT may be useful for target localization.

Target localization was studied using planar images from orthogonal detector views. The technical simplicity of planar imaging is appealing. There is no need for image reconstruction, and a set of images from a dual detector system might be used for localization or real time monitoring. However, results from the current study show limitations to this approach. On average, localization performance was worse using planar imaging as compared with either proximal 180° or 360° SPECT scans. In planar images, target positions were sometimes obscured by overlying and underlying activity from the background and the other spheres. At certain views, attenuation was nonuniform across the target, and especially so when viewing through the couch edge. These effects bias the apparent tumor location.

Our previous SPECT localization studies used simulated data.^(^ ^6^ ^,^ ^7^ ^)^ Scanner data are subject to imperfections in the detection process (e.g., finite detector response, limited energy resolution, septal penetration, nonuniformity, nonlinearity, and misalignment).^(^ ^25^ ^–^ ^27^ ^)^ Because of these effects, it was necessary to evaluate localization performance using real scanner data. In contrast with our previous computer‐simulation studies where the true target positions can be known exactly, true target positions were estimated in this study using CT and 12 hr SPECT images. Accurate estimates of the true target positions were important for subsequent assessments of localization errors. Residual errors in these position estimates were smaller than the width of CT voxels and were vastly smaller than SPECT spatial resolution, which in projection images was about 6.5 mm FWHM at 10 cm distance from the LEUR collimator used in this study.

Target localization was analyzed with respect to iteration number and postreconstruction smoothing. The effects of iteration number and smoothing were most noticeable for distal 180° trajectories where target localization was poor, but these trajectories are not likely to be used clinically. For targets with mean localization errors ≤ 2 mm, there were many outliers from the first iteration, suggesting that more than one image update is needed. Differences in the maximum localization errors were > 1 mm between the best and worst combinations of smoothing and iteration number in several cases. That noted, the standard deviations of the mean localization errors ranged from 0.11 to 0.28 mm when using proximal 180° trajectories, suggesting that localization performance typically would not be degraded substantially when using suboptimal parameter values, as likely would be the case clinically.

Target localization was studied for three detector trajectories. Localization performance was either comparable or better using a proximal 180° detector trajectory as compared with a 360° orbit. Localization performance was the worst using a distal 180° trajectory. This rank order in localization performance is predicted by the attenuation values and the collimator‐target distances shown in Fig. 9 and reported in Table 1. These findings are consistent with cardiac SPECT studies comparing 180° and 360° trajectories.^(^ ^28^ ^,^ ^29^ ^)^ Note, however, that neither the left nor right 180° trajectory was optimized for specific target sites in this study. For instance, a posterior 180° trajectory would decrease the distance between target B and the collimator while increasing attenuation survival probability in comparison with the three investigated detector trajectories. Based on trends discussed above, it follows that localization of B might be improved using a trajectory other than those considered here. These findings support the use of site specific, proximal 180° detector trajectories for imaging of superficial targets with parallel‐hole SPECT. For an individual patient, a detector trajectory might be optimized using treatment planning images to determine 180° trajectories that minimize the distance to and attenuation of a target.

This study shows that parallel‐hole SPECT is better for localizing superficial targets than deep targets. The volumes of deep targets C and D were 1.8 to 3.7 times larger than superficial targets A, B, and E; however, mean localization errors of the deep targets were on average approximately twice that of superficial targets in images from proximal 180° trajectories. The challenge of imaging deep targets may be addressed with converging collimation.^(^ ^30^ ^)^

Attenuation correction typically improved localization performance; however, for the well‐localized targets, the improvements were 0.15 mm, on average. The clinical benefit of improving localization by this amount is unclear. It may be that in certain scenarios, SPECT can provide useful information for target localization without corrections for attenuation.

## V. CONCLUSIONS

Target localization was studied in scanner‐acquired planar and SPECT images of a whole‐body phantom. Localization was generally worse using planar images as compared with proximal 180° and 360° SPECT scans. Using parallel‐hole SPECT, localization errors were substantially smaller for the superficial targets than the deep targets. Attenuation correction typically reduced localization errors, but for the well‐localized targets, target proximity to the detector trajectory generally had a larger effect. Localization performance was found to be robust to iteration number and smoothing. Using a proximal detector trajectory and attenuation correction, localization errors were within 2 mm for the three superficial targets, thus supporting the current role in biopsy and surgery, and demonstrating the potential for imaging inside radiation therapy treatment rooms.

## ACKNOWLEDGMENTS

This project was supported in part by the Department of Defense, Breast Cancer Research Program — predoctoral traineeship W81XWH‐08‐1‐0365. The content is solely the responsibility of the authors and does not necessarily represent the official views of the Department of Defense. The authors would like to thank Dr. Ron Jaszczak for use of the SPECT scanner and Mr. Kim Greer for his assistance with data collection.
